# Criminalistic and criminological analysis of the Thomas Restobar case in Peru in the context of sanitary measures by COVID-19: A case study

**DOI:** 10.12688/f1000research.125695.2

**Published:** 2023-09-12

**Authors:** José Antonio Jauregui-Montero, Oriana Rivera-Lozada, Carlos Neyra-Rivera, Cesar Antonio Bonilla-Asalde

**Affiliations:** 1 Postgrado, Universidad Norbert Wiener, Lima, Lima, Lima 32, Peru; 2 South American Center for Education and Research in Public Health, Universidad Norbert Wiener, Lima, Lima, Peru., Universidad Norbert Wiener, Lima, lima, Lima 32, Peru

**Keywords:** Criminology; Legislation; Criminal law; Social and economic law.

## Abstract

**Background:** The aim of this study was to collect and analyze the experiences in regard to the application of criminalistic and criminological protocols and procedures, which were used to carry out the intervention in the premises of Thomas Restobar in Peru, in the context of the declaration of sanitary measures to control COVID 19. This police intervention resulted in the death of 13 people.

**Methods:** For the collection of information, we used the focus group technique, for which a script was designed and validated by five experts, considering six major subcategories: C1SC1: Joint investigation and prosecution work, C1SC2: Protocols and guidelines, C1SC3: Chain of custody (police, experts, and prosecutor), C1SC4: Quality of results; C2SC1: Participation of agents of Thomas Restobar and the municipality, C2SC2: Compliance with DIGESA-DIRIS health regulations. The study was approved by the Institutional Research Ethics Committee of the Universidad Norbert Wiener, with approval file N°864-2021.

**Results:** From the criminological analysis it became evident that the deficiencies identified in the six structured subcategories have led to the tragic death of thirteen people from asphyxiation. The people who attended this bar did not comply with the sanitary norms, exposing the health and life of their relatives and other people. The security agents of the premises and of the Municipality of Los Olivos did not comply with the norms of supervision and control, which finally led to the unfortunate death of 13 people from asphyxiation, closely related to the case of Utopia, in 2002.

**Conclusions:** When evaluated by the experts of the focus group, it has generated two emerging categories: creation of a School of Experts and the categorization of the experience, which would prevent cases like Utopia and Thomas Restobar from happening again.

## Introduction

On August 22, 2020, approximately at nine o'clock at night, the main press, written, radio and television media in Peru, reported that an event took place in the Thomas Restobar discotheque in the city of Lima, capital of the country, with a massive attendance of people, where as a result of a police intervention in the context of the health emergency due to COVID-19, thirteen people died.

In Peru, the government established a compulsory curfew from 22:00 to 04:00, in addition to the prohibition of events involving crowds of people in order to avoid an increase in cases of COVID-19 in the country. However, the managers of “Thomas Restobar”, located in the district of Los Olivos, ignored the above provisions and organized a clandestine party. It resulted in the death of 13 people from asphyxiation as the more than 120 attendees tried to escape from the premises to avoid being arrested by the police ( Gálvez, 2020). This information is consistent with what was published by the newspaper El Comercio (2020). According to their reports, it was the first major tragedy in the midst of the coronavirus pandemic, at least in Latin America, which was caused by the fact that people did not to follow the restrictions imposed. Thus, it was one of the aspects considered in this research as the cause of the tragedy. However, it was not the only festive activity in which the police intervened in the country, as in the northern city of Chiclayo, the day before the tragedy in Lima, the officers detained 40 people; and a week before, the police intervened with 90 people in another area of the city where people drank alcohol and danced in the premises of a transport company, without taking into consideration the sanitary control measures, although there were no deaths as was the particular case of Thomas Restobar.

Three emblematic cases were analyzed: Case Utopia in Peru, Discotheque “Kiss” in Brazil, and clandestine premises in Spain.

We aim to examine the action protocols of the criminalistic and criminological investigation, identifying the relationship that was established between the institutions in charge of the investigative procedural function (Intitute of Legal Medicine, National Police of Peru [PNP], General Directorate of Environmental Health - Digesa and Municipality). Also, we describe the participation of the professional criminalistic experts in terms of compliance with the protocols and procedures, assessing the opinion that other professionals have regarding their compliance, and whether or not they were up to the circumstances. In addition, this research aims to identify the legislative, regulatory and operational factors that caused it; to make them known so that these unfortunate events, which have caused so much physical, psychological and health damage in the family and community environment, are not repeated.

The above mentioned, together with the new demands about the processes to guarantee the quality of the procedures, make it necessary to carry out a study to generate evidence about the role played by the compliance with norms, protocols, criminalistic and sanitary procedures, at the starting point of an emblematic case of media repercussion such as what happened in the present case of investigation.

This study will contribute to generate a perspective to be taken into account by the operators of the law, in regard to people’s actions with respect to the State, related to avoidable issues, which has to allow us to establish a change that should be based on the lessons learned, in order to ensure the protection of the fundamental rights of people.

It is too early to judge the impact of the COVID-19 pandemic in the country, in the context of the declaration of the emergency situation, however, even without further evidence, interesting lessons learned in the health, economic, social, political and legal fields can be identified, and this case is one of them.

Therefore, the questions that arise are: why did the deaths of these people occur; were there deficiencies in the application of operational plans and action protocols, as well as irresponsibility and/or negligence on the part of the various actors involved; and were there deficiencies in the application of operational plans and action protocols? For this reason, the aim of the study is to investigate the perceptions of the actors regarding administrative and criminal responsibilities. The objective of the study is to analyze and interpret the experiences on the application of criminalistic and criminological protocols and procedures used in the investigation of the Thomas Restobar case in Peru in the context of the declaration of sanitary measures by COVID-19.

## Literature review

The PNP’s mission is to: “…provide protection and assistance to individuals and the community, (…) investigate and combat crime (…) within the framework of a culture of peace and respect for human rights” (National Police of Peru, 2019, para. 1). To achieve this functional activity, the Directorate of Criminalistics of the National Police of Peru was created as a governing body of the Police Criminalistic System, with functional, administrative, regulatory, operational, technical, and scientific competence. It provides support to police units in the development of their functional activities by conducting technical and scientific tests, expressing opinions, issuing reports and other official documents at the request of the competent authorities as part of its functional activity established in the current legal regulations ( Dirgen-PNP, 2018).

In compliance with this regulation, the first police officer who arrives at the scene is who, after verifying the existence of victims in order to help, will proceed to provide protection and isolate the scene, guaranteeing that nobody trespasses, using any suitable mechanism for their demarcation or cordoning (plastic tape, cords, etc.), adequately defining their limits and establishing the ways in and out of the isolated scene. If necessary, a double perimeter may be established ( Minjus, 2014, p. 51).

In 2014, the New Code of Criminal Procedure was implemented, as well as the Regulations of the Chain of Custody. It aims to establish and unify basic procedures and responsibilities of the representatives of the Public Prosecutor's Office and officials, in order to ensure the authenticity and preservation of the material elements and evidence incorporated in any investigation of a punishable act, aided by forensic sciences, criminalistics, among other disciplines and techniques that serve the criminal investigation. In addition, it also aims to unify the general guidelines for the security and conservation of seized goods ( MP-FN, 2006).

The culture of quality in the criminalistic function in Peru has not yet prospered because it is a voluntary procedure and due to its high implementation cost. Experiences such as the Quality Management Systems (QMS), in Argentina, in forensic laboratories are only known in the field of medicine, and only in specific cases, such as genetic analysis laboratories, […] the implementation of quality systems in forensic laboratories is increasingly in high demand, and constitutes a strategy for the continuous search for the quality of results, trying to minimize the margins of error ( info-Lab Laboratory, 2020). In addition, we should note that the National Institute of Quality-Inacal, in Peru, aims to promote and ensure compliance with the National Policy for Quality in order to enhance the development and competitiveness of economic activities and consumer protection. Additionally, Inacal is the institution entrusted by the State to guarantee quality in such a way that companies obtain competitive advantages, by having laboratory services, certifications, and inspections based on integrity, efficiency and competence demonstrated with accreditation ( Ministerio de la Producción, 2016).

In the congress organized by the Crime Laboratory Division in Peru, the work of the first respondent and the main challenges in a digital forensic laboratory was presented. Through this presentation, it was remarked that, today, police officers should be prepared and be able to collect evidence, as it is likely to find security cameras, mobile devices, computer servers, and they should work on and lift these devices, which are indications and recommendations that should not be alien to a superior. In regard to the Restobar case, it was indicated that it was not clear what had happened with the video files or their destination. Loyola (2021), Which was confirmed when the corresponding expert reports were made.

Justice cannot be administered without the legal decisions determined by the Prosecutor and the Judge; therefore, we have taken into consideration the doctrines of Taruffo (2013) as this author mentioned that “Justice of the decision does not only presuppose its legality, that is, the derivation of a correct interpretation and application of the rules, but also truthfulness, this is, the inquiry of the truth about the relevant facts” (p. 98). In this regard, “The ultimate goal is to reach a stage in legal studies oriented by empirical research, in which it is possible to speak of evidence-based law” ( Páez, 2015, p. 148). Thus, the determination of the facts is based on the reasons’ acceptability and validity criteria in order to consolidate the theory of evidence and not the subjective situation of the judge.

For this reason, it is necessary to include the legal regulations prior to the implementation of COVID-19 sanitary measures in the analysis; thus, Article 292 of the Penal Code typifies the crime of violation of sanitary measures as follows:

“Whoever violates the measures imposed by law or by the authority for the introduction into the country or the spread of a disease or epidemic or an epizootic or plague, shall be punished with imprisonment of not less than six months nor more than three years and a ninety to one hundred and eighty days-fine” (Diario Oficial El Peruano, 2020).

The World Health Organization, on March 11, 2020, declared the SARS-CoV- 2 coronavirus outbreak (COVID-19) to be a pandemic. For this reason and in accordance with its constitutional competence, the Peruvian Government approved Article 1 of the Supreme Decree N.°020-2020-SA, which refers that: “The sanitary emergency declared by Supreme Decree N° 008-2020-SA is extended from June 10, 2020 for a period of ninety (90) calendar days” ( Presidency of the Republic, 2020a).

Article 3 of the Supreme Decree N.°139-2020-PCM, norms about the limitation of the exercise of the right to freedom of transit of persons during the State of National Emergency, established the mandatory curfew of all people from 22:00 to 04:00 of the following day, from Monday to Saturday nationwide ( Presidency of the Republic, 2020b).

Regarding the functions of the municipalities during the sanitary emergency and with respect to their competence to control the freedom of transit of persons in the context of the Declaration of State of Emergency that imply the restriction of the exercise of constitutional rights related to personal freedom and security, inviolability of the home, and freedom of assembly and transit in the territory, as is the case of what is established in S.D. 008-2021-PCM (2021), the competent entities that are in charge of executing such measures are the PNP, with the support of the Armed Forces (FF.AA.). They ensure compliance with the provisions of the regulation of the state of emergency, for which purpose they can verify and intervene persons, goods, vehicles, premises, and establishments that are necessary to verify and, if necessary, prevent the carrying out of services and activities that are not allowed. They exercise control with respect to the limitation of the exercise of freedom of transit at the national level. The municipalities are obliged to collaborate with the PNP in this task.

In particular, S. D. 008-2021-PCM (2021) establishes that: “Local authorities have the duty to collaborate and not hinder the work of the police and military authorities in the exercise of their functions. In addition, local governments contribute (…), within the framework of their competences”. ( Gob.pe, 2020a)

In regard to the functions of municipalities during the health emergency regarding public and private spaces in the case of activities or events that involve the concentration of people in indoor or outdoor spaces that offer great risks for the transmission of COVID-19, municipalities should contribute with the dissemination of information and sensitization of neighbors in order to avoid the organization or participation in activities that involve the agglomeration of people. In case of identifying places where these activities take place, municipalities should coordinate with the PNP to intervene, as the Ministry of the Interior (Mininter) is the competent entity to provide authorization for mass events. In addition, the entity itself should coordinate the measures of control of activities or events that involve the concentration of people in indoor and outdoor spaces that pose risks of COVID-19 transmissibility, and ensure that all commercial establishments and markets help in prevention efforts ( Gob.pe, 2020b).

## Methods

Research with a qualitative approach was developed, with an intrinsic case study design, which presents its own specificities, “(…) aiming to achieve a better understanding of the specific case to be studied (…) the case is not chosen because it is representative of other cases, or because it illustrates a certain problem or feature, but because the case itself is of interest” ( Stake, 2005, cited in Jiménez Chaves & Cornelio Comet, 2016, p. 7).

The primary sources of information used came from seven Peruvian professional experts, students of the Master's degree in Criminal Sciences of a Peruvian University, selected incidentally: a police crime scene expert, a biologist, a psychologist of the Ministry of Women, a lawyer Social Specialist of the Ministry of Women and Vulnerable Populations, two prosecutors of the Public Ministry, a lawyer of the Superior Court of Specialized Criminal Justice.

The authors did not have a previous relationship with the experts participating in the focus group. The experts were informed about the objectives of the study prior to their participation and the signing of the informed consent.

For the collection of information, the focus group technique was used, for which a script was designed and validated by five experts in law, for this purpose six open questions were applied, so that the experts could express themselves without limitations in their answers. For the development of the focus group, the sanitary provisions in force by COVID-19 were taken into consideration, being carried out virtually on the Microsoft Teams platform, being recorded in audio and video to be transcribed and evaluated.

Before the execution of the focus group, the researchers met three times to clarify the purposes of the activity and to review the content of the guide and to familiarize themselves with the open questions related to the objectives of the protocol. Additionally, it is important to mention that the researchers have experience in conducting qualitative research and the use of the focus group technique; the duration of the focus group session was two hours.

The transcripts were not given to the participating experts for their comments and/or corrections, they were only analyzed by the researchers.

The focus group participants have been coded as E1: police crime scene expert; E2: lawyer; E3: lawyer; E4: biologist; E5: prosecutor1; E6: prosecutor2; E7: psychologist and their responses RE1, RE2, RE3, RE4, RE5, RE6 and RE7.

The study was approved by the Institutional Research Ethics Committee of the Norbert Wiener University, with approval file No. 864-2021. The study participants gave their written informed consent and their identity was protected. The purpose of the research was to analyze from the perspective of important stakeholders – through their perception and opinion – to provide fundamental information about the object of study. Hence the usefulness of this approach for the study of what happened in Lima at Thomas Restobar in the context of the declaration of sanitary measures by COVID-19.

It was convenient to look at what happened from different angles to improve the perspective of the norms, protocols and procedures; as well as the conduct of the agents involved in this public event. In such sequence of ideas, the case study that was used in the to the criminalistic investigation ( Jiménez and Comet, 2016, para. 6).

Finally, in the triangulation, we proceeded to contrast and compare the focus group data, to identify coincidences and differences, incorporating symbolic interactionism, through a sociocultural interpretation, starting from the perception from the media point of view, which can influence the opinion of the actors involved in the study allowing to study in an economical, simple and undemanding way, the reality that surrounded about the methodological process of the study.

In Table 1 we can see the two categories: C1-Analysis of criminalistic procedures (1. Joint investigation and prosecution work, 2. Protocols and guidelines, 3. Chain of custody, 4. Quality of results); and C2-Criminological analysis and COVID-19 sanitary measures (1. Participation of Thomas Restobar agents and the municipality, 2. Compliance with DIGESA-DIRIS sanitary regulations), with four and two subcategories, respectively.

**Table 1.** Categorization table of the study.

Category	Subcategory
C1-Analysis of criminalistic procedures	1. Joint investigation and prosecution work 2. Protocols and guidelines 3. Chain of custody 4. Quality of results
C2-Criminological analysis and Covid 19 sanitary measures	5. Participation of key players of Thomas Restobar and the municipality. 6. Compliance with DIGESA-DIRIS sanitary regulations

## Results

The results have been organized into two categories and six subcategories, with the additional identification of a meta-category called “Systematization and socialization of the experience” that synthesizes and groups the two categories: C1-Analysis of criminalistic procedures and C2-Criminological analysis and COVID-19 sanitary measures.

### Category 1 Analysis of criminalistic procedures: Subcategory 1 Joint investigation and prosecution work

The main objective of the criminalistic procedures is to allow the formalization of the prosecutor's report, so that the criminal process can be supported by scientific and irrefutable evidence.

*Question: What are the advantages and disadvantages of the double participation of the national police and the Public Ministry in the criminalistic procedures and the issuance of expert reports, which will determine the formalization of the prosecutor's accusation?*

RE1, RE7 state that “…it is an advantage to have two institutions that look at the criminalistic aspect, because the police do not meet certain standards, and the Public Ministry does not have the number of personnel to deal with a crime scene……” This is shared by RE2.

According to RE2 “… there should be an organization, an entity that can bring together the 2 entities of both the National Police and the Public Ministry to be in charge of the quality of the criminal investigation”.

Continuing the analysis, RE3, RE5, RE6 state that given the administrative difficulties that exist between the National Police and the Public Ministry; perhaps it would be good to give this attribution to a private entity but the worrying thing is that it could generate a lot of suspicion, we have seen it with corruption issues, as an example the Odebrecht case and other institutions, in that same reasoning according to RE6 “it is necessary to create a school of experts, but the state has to control it, it may work…”

Delimitation: The joint investigation and prosecution work is an advantage because they complement each other, it is a team effort. Weaknesses were identified such as: lack of personnel at the local and national level, hindering the formalization of the prosecution report; there is a lack of a State supervisory body to facilitate the work of both institutions.

### Category 1 Analysis of criminalistic procedures: Subcategory 2 Protocols and guidelines

The main objective of the protocols and guidelines is to ensure the integrity and quality of the processes.

*Question: What is your opinion regarding the Thomas Restobar case and the performance of the institutions involved in the timeliness, application, processing and chain of custody of the police, criminalistic and Public Ministry protocols?*

The dialogue established during the development of the focus group has highlighted the experiences of the experts in the handling of the protocols and guidelines for the formalization of the public denunciation, which could be useful to understand their value in the analysis of what happened in Thomas Restobar Lima-Peru.

RE1 explains “…we have seen that the first level of protection of the place of the facts was violated by the police officers, because they stole the DVR (Digital Video Recorder, the interpretation of the acronym is of the investigators), apparently with the content of the video images…”

RE2, RE3, RE4 point out “…. yes there are these protocols of action to intervene in these investigations, but they are not easily accessible; there should be a portal to disseminate and publicize all these procedures, in a single portal. RE3 complements “…… should have acted with the corresponding police protocols, they were not organized correctly, despite the fact that the police intervened in the end there were several deaths and all this is related to the “utopia case”, there was no compliance with the use of protocols, they acted on impulse and did not measure the consequences of their police intervention”. RE4, RE5, RE6 consider that “…these criminalistic and operative manuals, protocols exist, however, they are not being applied, we are not prepared at the organizational level, they have not adopted the corresponding preventive measures since I can say a phrase “We have not learned from the mistakes of the past (…) only when the facts happen we reflect on it”.

Delimitation: The police officers who intervened did not guarantee the intangibility of the scene; there are protocols, norms, guidelines, but they did not apply them correctly; they acted on impulse without measuring the consequences. They were not prepared; “We have not learned from past mistakes”; recalling the “Utopia” case where 23 people died.

### Category 1 Analysis of criminalistic procedures: Subcategory 3 Chain of custody (police, experts and prosecutor)

*Question: What is your opinion regarding the Thomas Restobar case and the performance of the institutions involved in the timeliness, application, processing and chain of custody of the police, criminalistic and Public Ministry protocols?*

Regarding the analysis of the chain of custody, the disappearance of the DVR and therefore of the images recorded in this equipment was noted. In this regard, RE1 states “…it is assumed that the first interveners should have protected this scene, they are the guardians of the elements of evidence, if from there, the chain of custody has already been violated; protecting, isolating the crime scene or the scene of the crime is already a chain of custody (…) I consider that neither Public Ministry nor the experts were aware of this violation, until they arrived at the place where this DVR was missing and they did not know if it had been the people who were at the place or if it had been the police, there was a question: Who would have stolen this DVR? If it had been filmed or not, how did the events occur? I believe that the facts came to light through the press, who were the ones who disseminated the first images that showed that the DVRs were indeed taken from the scene. Then I consider that they have not acted adequately…”. RE2, RE3 and RE6, state that “…. the first thing to see is the police intervention and then the analysis of the cameras that was later disseminated, this allowed to see the contradictions that had been given in the first versions of the case, and this has allowed to determine above all that the police protocols had not been complied with”. RE4, RE6, RE7 consider that “…a lot of evidence was also lost due to the number of people, the family members who attended, the neighbors, the protocols came to nothing, and everything was chaotic that day”. Regarding the same, RE5 states “…… there was not a good communication between the police and the representative of the Public Ministry, but I understand that the police immediately reported the event, however the Public Ministry arrived a little late, even though being part of the Public Ministry, I cannot defend the indefensible, when a Prosecutor is on duty, he is on duty 24 hours a day. In conclusion, what I want to say is that this issue of the chain of custody was not respected at all; likewise, some basic criminalistic procedures were not taken into account, as I say and sometimes I also mention that the principles of Criminalistics were not taken into account, perhaps to adequately approach this type of investigation, there was an incorrect synergy between the police and the representative of the Public Ministry.

Delimitation: There was an incorrect chain of custody procedure. There was an incorrect synergy between the police and the representative of the Public Ministry.

### Category 1 Analysis of criminalistic procedures: Subcategory 4 Quality of results

*Question: How are we prepared to guarantee the quality of the results of forensic procedures?*

Inacal and other institutions refer to the importance of the culture of quality of the results to guarantee the effectiveness and efficiency of these in the processes in general that are also applied in the criminalistic processes. Thus, in RE1 it is mentioned: “….. I believe that there has not been a quality of the results, I consider that it would be feasible to use a private company, because as I said before, the laboratory of the National Police nor the Public Ministry, do not have an ISO quality certification nor quality standards, so they could be questioned; in the analysis of the opinion of RE2 it is found that ”…. the quality of the procedures is related to training, infrastructure, implementation of the departments, investment in technology; there should be a unit to verify the procedures and really contribute to improve the work of the investigators; Inacal should intervene to ensure that these procedures are correct, thus RE4, RE7 agree with what was stated; thus RE5 also adds the high rotation of investigators, absence of a standardized format of expert reports, lack of a school of experts, more experts (….) all these aspects, since they are not taken into account become factors that weaken the quality of the results.”

RE3 states: “…it is required to be an expert to have enough experience in the matter (…) then in this case Thomás Restobar apparently has not been carried out correctly, it has been wanted to act properly; becoming evident that we are not prepared to guarantee the quality of the results, even though there are standard procedures these are not carried out"; within this same line of reasoning in RE6 also highlights that “…. … most of these experts have been trained by the National Police in different trainings, and their expertise makes them better, we need a National School of Experts as in other countries of the world”.

Delimitation: The National Police and the Public Ministry do not have ISO quality certification and quality standards. Inacal should intervene in order to improve the processes or a private institution that performs this function. Additionally, a national school of experts should be created.

### Category 2 Criminological analysis and COVID-19 sanitary measures: Subcategory 1 The participation of key social players of Thomas Restobar and the municipality

*Question: What is your opinion of the Thomas Restobar case and the actions of key social players of Thomas Restobar and the Municipality of Los Olivos?*

Regarding the participation of the key players of Thomas Restobar (the owner and the tenant of the premises responsible for the event’s organization), in the experts’ opinion, it is advisable to exclude them from the analysis, since there is an ongoing investigation by the Public Ministry to determine their responsibility.

Regarding the participation of the key social players of Thomas Restobar and the municipality, interviewees RE1, RE2, RE3, RE4, RE7 told us “……. that the preventive part is neglected, that they are responsible, that if we are in a state of emergency, the municipality is in charge of enforcing it, because that place should have been closed, why was it open at that time? They should have fulfilled their task of control and supervision of these businesses, it seems that a license had already been previously denied, so this place already had problems, it was a place that should have received more attention”; Finally, RE7 stated that “… we were in a state of emergency, what were these people doing at that time in this place, concentrated, not respecting the social distance”.

RE3 reports that “…it did not have the conditions for a restobar, for example, it had only one entrance and exit staircase, if an inspection had been carried out previously, which was not done, if the corresponding fine had been imposed to fix that aspect, I believe that so many victims would have been avoided, at the time the police entered. In addition, in RE5 he also reminds us that ”…… we are always going to relive the case of Utopia (discotheque where a fire occurred in 2002 and 13 people died - this explanation is from the investigators), the tragedy of that time could have been avoided, the municipality can establish sanctions, fines, temporary or permanent closure, but nothing was done”. Thus in RE6 he reminds us: “…… the mayor justifies his actions by indicating to the press that he did not have enough economic resources to hire more personnel, so I believe that here there are responsibilities on the part of the municipal authorities, well it is assumed that the death of any person produces in the first place serious consequences of sentimental order, patrimonial, he considered that it is necessary to foresee and prevent.

Delimitation: It is very strange that the officials of the municipality did not realize that it did not have the optimal conditions for its operation. The mayor justifies his actions by indicating that he does not have the economic resources to hire more inspectors. The death of any person has serious psychological and financial consequences.

### Category 2 Criminological analysis and COVID-19 sanitary measures: Subcategory 2 Compliance with DIGESA-DIRIS sanitary regulations

*Question: What is your opinion of the Thomas Restobar case and the actions of the Directorate of Integrated Health Networks Lima Norte-Diris and the Ministry of Health’s General Directorate of Environmental Health-Digesa?*

From the focus group analysis, RE1, RE2 and RE3 say that: “… the rapid tests were done, the handling of these bodies generated contagion both to the intervening personnel and the prosecutor; they could have been infected; they do not know if there was any follow-up to determine if the quarantine was complied with.

RE4 adds: “… evaluations should have been made not only to the participants of these parties, but also to those who lived around them. Also RE5 and RE7 refer that people should have been aware that, beyond exposing their lives, they were also exposing their relatives, because when going to this center, to the discotheque, the mandatory social distance was not respected, they were in contact, even people who had Covid-19 were identified, this is also a responsibility on their part, without any doubt, responsibility on the part of these institutions for not exercising greater control. RE6 states: “… well I am not going to repeat what my colleagues have already pointed out, the only thing I am going to say is that the Public Ministry has opened proceedings for three crimes: Culpable homicide, culpable injuries, failure to comply with sanitary measures. I consider that the preventive function has been carried out by both the Directorate of the Integrated Health Networks of Lima Norte and the General Directorate of Environmental Health, it was seen in the videos that all the people were being analyzed with the tests and it was precisely there that almost all of them were infected, because they had all been without social distancing.

Delimitation: The rapid tests were performed on the police officers and people who were detained; others left without taking the rapid tests. It is not known if they complied with the corresponding quarantine, they would have spread the contagion to other local people and family members.

Finally, a closing question was posed; from their perception:

*Question: What changes do you consider or should be implemented to improve criminalistic work, for example, training of criminal profilers, dynamic retrospective, forensic planimetric, or others?*

In this regard, RE7 said that “…investigation is limited by non-optimized investigation capabilities, for various reasons such as: lack of technology, accreditation, optimization, therefore there should be an area of *interinstitutional coordination* of criminal investigation, interdisciplinary. RE2 “… … above all, it is necessary to systematize the experience, standardize procedures at the level of all instances, many of these people have years doing specialized investigations, but they have not been able to organize, systematize, create a magazine where all experts can publish the cases they have carried out”.

RE1 “… … The crime scene expert is the one who directs, organizes, plans and the forensic photographer is part of his team, of course, I think there should be protocols for everything”.

Delimitation: Strengthening of criminalistics. Their experiences are not shared. Creation of a school of experts. Creation of an interinstitutional coordination area for interdisciplinary criminal investigation. It has generated a meta-category called “Systematization and socialization of the experience”.

## Discussion

In Peru, the government established mandatory social immobilization from 22:00 to 04:00 hours, in addition to the prohibition of events involving crowds of people in order to avoid an increase in cases of Covid-19 in the country. However, the managers of the local “Thomas Restobar” located in the district of Los Olivos ignored the above provisions and organized a clandestine party that resulted in the death of 13 people by asphyxiation, as the more than 120 attendees tried to escape from the establishment to avoid being arrested by the police ( Gálvez, 2020); This information is consistent with the information provided by the newspaper El Comercio (2020) that it is the first major tragedy in the midst of the coronavirus pandemic, at least in Latin America, due to the decision of the people not to follow the restrictions imposed; it will be one of the aspects to be considered in this research as the cause of the tragedy. However, it was not the only festive activity in which the police intervened in the country, in the northern city of Chiclayo the day before what happened in Lima, the agents arrested 40 people. A week before, the police intervened 90 people in another area of Lima where they drank liquor and danced in the premises of a transport company, without taking into consideration the sanitary control measures; although there were no human losses to regret; as was the particular case of the study.

Three emblematic cases will be taken to analyze: Utopia case in Peru, Discotheque “Kiss” in Brazil and a clandestine premises in Spain.

A fire in the discotheque Utopia-Peru occurred on July 20, 2002; it had a tragic end with 28 dead and 57 injured. This place had no operating license because it did not comply with the minimum standards of civil defense. People tried to flee, but it was difficult for them to leave, because there were no signs indicating the emergency doors ( EnfoqueDerecho.com, 2022). Also on January 23, 2013, in a fire at the Kiss nightclub in southern Brazil, 242 people died; there was only one exit and there were scenes of panic as people tried to get out ( BBC, 2013). In both cases the unfortunate end was due to having only one escape door and security deficiencies.

In the case reported by the newspaper La Vanguardia (2020) where the Barcelona Police intervened 58 people in a clandestine party without masks in an event in the context of the COVID-19 pandemic, no deaths were recorded; note that in this case and other irregular events that occurred in other places in Peru did not result in deaths to regret; we believe that the concurrence of deficiencies that we will find in this research did not occur.

The following, based on the experiences of these emblematic cases, we will analyze the categories identified in the light of the findings of this study, from the perspective of the experts interviewed.

The category of joint work between the National Police and the Public Ministry is regulated and established to exercise interinstitutional coordination functions protected by the Political Constitution of Peru, its organic laws, the Code of Criminal Procedures, protocols of the Ministry of Justice and other provisions, in order to achieve an efficient function. The Ombudsman (2020) in a letter sent to the President of the Republic, asks the Government, together with local authorities, coordination with neighbors and the National Police of Peru, to contribute to the surveillance and prevention of mass gatherings of people during the COVID-19 pandemic. The experts have perceived deficiencies in the intervention process, which could be associated to the death of the 13 people by asphyxiation; evidenced through the videos spread by the press and those recorded by the security cameras of the premises.

The category of protocols and procedures of action, allows standardizing and ensuring the efficiency and effectiveness of the procedures; in our country the Supreme Decree 010-2018-JUS (2018) establishes that the first police officer who arrives at the scene must proceed to its protection and isolation guaranteeing its intangibility; adequately defining its limits and establishing the routes of entry and exit of the isolated scene; we have been able to evidence that there are; for example the Protocol of interinstitutional action for the protection and investigation of the crime scene, the Protocol of specific interinstitutional action for the protection and investigation of the crime scene. In this regard Taruffo (2013) and Páez (2015) agree that the determination of facts is based on criteria of acceptability and validity of reasons in order to consolidate the theory of evidence, guided by empirical research, in which it is possible to speak of evidence-based law. In the opinion of the experts, the manner in which the police approached the only entrance and exit door, closing it, caused the people trying to leave to crowd the stairs, which was evidenced in the video images disseminated by the press and the security cameras of the premises. This made it possible to determine that an operational plan of intervention, approach and processing of the place of investigation was not applied, in spite of having the respective regulations and protocols in place.

The Chain of Custody category starts with the participation of the first police intervener; it is fully regulated in the Code of Criminal Procedure; the Guide of Criminalistic Procedures of the National Police of Peru (PNP), the Manual of Criminalistic Expert Procedures; Loyola (2020) warns that a superior cannot be unaware that it is very likely that mobile devices, security cameras, servers, computers are found; regarding the case, he believes that more than six thousand video files were recovered or wanted to be deleted; it would have to be seen if these files were deleted on the day or at the moment when the agent was intervening. In the opinion of the experts, the chain of custody procedure was incorrect because there was an inadequate synergy between the police and the representative of the Public Ministry, this process was also flawed; this was also seen preliminarily in the video images disseminated by various media and later corroborated by the forensic experts when processing the scene, finding that the video recording equipment - DVR - was not in place. Therefore, it is evident that not only the chain of custody was not complied with, but also that an attempt was made to hide evidence, whose purpose is of probative value in the investigation.

The category quality of the results seeks the truth of the facts, which in the theory of evidence is about the criteria of acceptability and validity of the reasons, not the subjective situation of the Judge. In this context, the Ministry of Production counts on the National Institute of Quality (Inacal) to guarantee that the processes are executed in compliance with the protocols. Info-Lab Laboratory (2020) states that the implementation of quality systems in forensic laboratories is increasingly demanded, and constitutes a strategy for the continuous search of the quality of the results, trying to minimize the margins of error. The results obtained showed that there was no quality, that the laboratories of the National Police and the Public Ministry do not have an ISO 17025 quality certification, and recommended that it would be feasible to use a private company or that Inacal would have to intervene to ensure that these procedures are correct and above all that there is uniformity of procedures, as well as to promote the development and awareness of the institutional “culture of quality”.

Regarding the category participation of players of Thomas Restobar and the municipality, the Organic Law of the municipalities establishes its supervisory role, granting licenses and sanctions, it should contribute with the dissemination and sensitization of neighbors in order to prevent them from organizing and participating in activities that involve the agglomeration of people; Agencia Andina (2020) also agrees that it should prohibit social gatherings in bars and nightclubs during the health emergency, taking into consideration that the civil defense certificate of the premises would have expired in January 2020 . Cahuana (2020) agrees with the Ombudsman Office (2020) on the participation of the organizer of the event before an administrative responsibility, for organizing this type of events in a place that was not adequate. The experts are of the opinion that it is evident that the municipality has not fulfilled its supervisory role, as well as the preventive work that it is responsible for, considering also co-responsibility with the owner and the administrators of the premises; emphasizing also that the death of any person produces in the first place serious consequences of psychological and patrimonial order.

Regarding the category of compliance with DIGESA-DIRIS sanitary regulations, article 292 of the Penal Code typifies the crime of violation of sanitary measures, article 3 of the Supreme Decree No. 139-2020-PCM (2020) provides for the mandatory social immobilization of all persons in their homes from 22:00 hours to 04: Pacheco (2020) confirms that necropsies were performed on the 13 bodies and that rapid tests were applied to the police officers who intervened, but it is not known if the people who tested positive have complied with the respective quarantine; In the opinion of the experts, the Digesa-Diris would have complied with the preventive function by disseminating the sanitary dispositions; however, many people withdrew without being tested and all those who tested positive were not informed of their compliance with the quarantine, which may have determined that the contagions occurred to the family members and their environment.

## Conclusions

The need to investigate and reflect on the advantages and difficulties involved in complying with the rules and protocols under the perspective of justice operators in sanitary conditions, as in the COVID-19 pandemic; has allowed the identification of six subcategories: Joint investigative and prosecutorial work; the protocols and action guides; the chain of custody; the quality of the results; the participation of the Thomas Restobar actors in charge of organizing the event and the municipality; compliance with DIGESA-DIRIS sanitary regulations; in all of them deficiencies have been established. As a result of the theorization delimitations and contrasts, a new emerging meta-category called “Systematization and socialization of the experience” was generated. Society is exposed to various events, due to its own irresponsibility or negligence on the part of the State, which can lead to tragic endings such as the case studied. In this sense, the judicial and health systems face challenges in order to correct and develop theoretical, methodological and attitudinal capacities, which allow the planning of actions and coordinated work, as quality instruments; in order to avoid physical, psychological and social damage in the family and community environment.

The need to investigate and reflect through a qualitative approach on the criminological and criminalistic factors that led to the unfortunate deaths of more than a dozen people at the establishment named Thomas Restobar in Lima, Peru, during a state of social lockdown decreed by the national government amid the COVID-19 pandemic, has allowed the identification of advantages and difficulties entailed in adhering to rules and protocols from the perspective of justice operators under sanitary conditions. This has contributed to recognizing six subcategories: Collaborative investigative and processing work; guidelines and protocols; chain of custody; quality of results; participation of key actors from Thomas Restobar and the municipality; compliance with health standards from DIGESA-DIRIS; deficiencies have been observed in all of them. In the first subcategory, there was inadequate coordination between the police and the prosecutor, as the prosecutor arrived long after the police intervention. The second subcategory reveals that guidelines and protocols exist but are not properly followed by police and prosecutors, who consider experience to be sufficient, thus sidelining the use of these tools. The third subcategory demonstrates a violation with the removal of video recordings from inside the Thomas Restobar discotheque, indicating that the protection of evidentiary elements falls under the responsibility of the police personnel. The fourth subcategory unveils weaknesses that compromise the quality of results, such as ongoing training, certification of experts, accreditation of forensic laboratories by recognized institutions, absence of a professional association of experts, adequate infrastructure, sufficient personnel, cutting-edge equipment and technology, among the most relevant issues. Concerning the fifth subcategory, the participation of key actors from Thomas Restobar was excluded from the analysis due to their involvement in an ongoing fiscal investigation. Regarding the municipality, the lack of preventive intervention is notable, given their role in oversight and granting operational licenses. Finally, in the sixth subcategory, it can be inferred that health standards were not met, exposing participants and, by extension, those in close contact to the risk of COVID-19 infection. Following the studied event, DIRIS conducted rapid tests, but the number of people tested, the number of positive cases, and whether these individuals were placed under quarantine remain unknown. Additionally, there is a probability that some attendees could have been infected, putting their close contacts at risk as well.

As a result of the delineations and contrasts in the subcategories, a new emerging metacategory was generated, named “Systematization and Socialization of the Experience.” It is understood that the experience of experts (both active and retired) should be considered as a source of consultation in political and academic decision-making. This also involves the establishment of a National School of Experts, a Professional Association of Experts, an inter-institutional Coordination Body responsible for criminal investigation, as well as seeking accreditation for forensic laboratories and the presence of nationally and/or internationally reputable accrediting institutions.

Society is exposed to various events, driven by its own irresponsibility or negligence and the inadequate response of the State, which can result in tragic outcomes, as demonstrated by the case under study. In this regard, judicial and healthcare systems face challenges in rectifying and developing theoretical, methodological, and attitudinal capacities that enable the planning of coordinated actions as quality instruments. The goal is to prevent physical, psychological, and social harm within the family and community context due to endemics such as COVID-19.

## Data Availability

Zenodo: Criminalistic and criminological analysis of the Thomas restobar case in Peru in the context of sanitary measures by covid 19: case study, https://doi.org/10.5281/zenodo.7030306 (Cesar Antonio bonilla-Asalde, Oriana Rivera-Lozada, José Jauregui-Montero, & Carlos Neyra-Rivera, 2022). Data are available under the terms of the Creative Commons Attribution 4.0 International license (CC-BY 4.0).
