# Supplementary material for: Criminalistic and criminological analysis of the Thomas Restobar case in Peru in the context of sanitary measures by COVID-19: A case study
Source: F1000Res. 2023 Sep 12;11:1356. Originally published 2022 Nov 21. [Version 2] doi: 10.12688/f1000research.125695.2 (PMC11109691; doi:10.12688/f1000research.125695.2)
Supplement: Peer review history

# Reviewer report: Reviewer response for version 2

**DOI:** 10.5256/f1000research.155271.r265772

**Published:** 2024-05-14

**Authors:** Isabel Menacho Vargas

**Affiliations:** 1 Universidad Nacional Mayor de San Marcos, Lima District, Lima Region, Peru

After conducting a thorough review of the manuscript titled "Criminalistic and criminological analysis of the Thomas Restobar case in Peru in the context of sanitary measures by COVID-19: A case study," I can affirm that it makes a notable contribution to the field of criminalistics and criminology, specifically regarding the implementation of protocols and procedures during the intervention at Thomas Restobar in Peru amidst the sanitary measures to contain the spread of COVID-19.

 The manuscript's structure is impeccable, with an introduction that not only clearly presents the research problem but also effectively captures the reader's interest. Furthermore, the theoretical review provides a solid and updated context, significantly supporting the study's relevance.

 The methodology employed is rigorous and appropriate for the type of study conducted, demonstrating a clear mastery of the qualitative techniques used in the case analysis. The inclusion of a coherent and detailed categorization matrix facilitates understanding of the methodological process and ensures the study's replicability.

 The results obtained are clear, concise, and relevant, reflecting the success of the employed methodological approach. These results not only contribute to the existing body of knowledge in the field of criminalistics and criminology but also offer valuable information for future research in this area.

 The discussion conducted demonstrates a deep analysis of the results, effectively contrasting them with previous works and similar experiences. This approach significantly enriches the article's quality, providing a more comprehensive and reflective perspective on the findings presented.

 The conclusions are robust and adequately supported by the results obtained, reinforcing the study's contribution to the field of study in question. Additionally, the inclusion of updated and pertinent references demonstrates a commitment to academic excellence and scientific integrity.

 In summary, this article not only meets the quality standards of our journal but also represents a significant contribution to the field of criminalistics and criminology, standing out for its methodological rigor, clarity of exposition, and thematic relevance.

 Finally, the article is APPROVED

Is the work clearly and accurately presented and does it cite the current literature?

Yes

If applicable, is the statistical analysis and its interpretation appropriate?

Yes

Are all the source data underlying the results available to ensure full reproducibility?

Yes

Is the study design appropriate and is the work technically sound?

Yes

Are the conclusions drawn adequately supported by the results?

Yes

Are sufficient details of methods and analysis provided to allow replication by others?

Yes

Reviewer Expertise:

Publication of scientific articles in indexed journals Scopus, WOS and Scielo in areas of psychology and social sciences, principal researcher in research projects funded by Peruvian universities, inventor and manager of innovations with the presentation of patents, copyright works and technological packages

I confirm that I have read this submission and believe that I have an appropriate level of expertise to confirm that it is of an acceptable scientific standard.

# Reviewer report: Reviewer response for version 2

**DOI:** 10.5256/f1000research.155271.r205640

**Published:** 2023-10-05

**Authors:** Effi Lambropoulou

**Affiliations:** 1 Panteion University of Economic and Social Sciences, Athens, Greece

Everything is fine with the article.

Is the work clearly and accurately presented and does it cite the current literature?

Yes

If applicable, is the statistical analysis and its interpretation appropriate?

Not applicable

Are all the source data underlying the results available to ensure full reproducibility?

Yes

Is the study design appropriate and is the work technically sound?

Yes

Are the conclusions drawn adequately supported by the results?

Partly

Are sufficient details of methods and analysis provided to allow replication by others?

Yes

Reviewer Expertise:

Criminology, Sociology

I confirm that I have read this submission and believe that I have an appropriate level of expertise to confirm that it is of an acceptable scientific standard.

# Reviewer report: Reviewer response for version 1

**DOI:** 10.5256/f1000research.138030.r177574

**Published:** 2023-06-22

**Authors:** Effi Lambropoulou

**Affiliations:** 1 Panteion University of Economic and Social Sciences, Athens, Greece

- The manuscript contains new information to justify publication.
- The title is adequate.
- The Abstract/Summary and the material are suitable.
- In general, the study is clearly and accurately presented. The text is written in good English and is easily understood.
- The used literature is technical and there is no theoretical context in the article; I don’t think that the authors had an intension of theorising.
- I cannot answer if adequate reference is made to other work in the field for the reasons mentioned above (technical).
- The details of methods are sufficient. The replication is difficult because the researchers have used focus groups and the same groups cannot be recreated.
- The study is appropriate for indexing after some changes: I could not find the analysis of the three other cases the authors say they compare (Case Utopia in Peru, Discotheque “Kiss” in Brazil, and clandestine premises in Spain). The cases are only referred twice, pp. 3 and 10.
- All the source data underlying the results are available to ensure full reproducibility.
- None of the material can be deleted without detriment.
- The conclusions drawn are adequately supported but not sufficiently.

Is the work clearly and accurately presented and does it cite the current literature?

Yes

If applicable, is the statistical analysis and its interpretation appropriate?

Not applicable

Are all the source data underlying the results available to ensure full reproducibility?

Yes

Is the study design appropriate and is the work technically sound?

Yes

Are the conclusions drawn adequately supported by the results?

Partly

Are sufficient details of methods and analysis provided to allow replication by others?

Yes

Reviewer Expertise:

Criminology, Sociology

I confirm that I have read this submission and believe that I have an appropriate level of expertise to confirm that it is of an acceptable scientific standard, however I have significant reservations, as outlined above.
